# A forced swim-based rat model of premenstrual depression: effects of hormonal changes and drug intervention

**DOI:** 10.18632/aging.202249

**Published:** 2020-11-21

**Authors:** Sheng Wei, Xiwen Geng, Zifa Li, Kaiyong Xu, Minghui Hu, Hongyun Wu, Wei Shi, Mingqi Qiao

**Affiliations:** 1 Department of Neurosurgery, Qilu Hospital of Shandong University and Brain Science Research Institute, Shandong University, Ji’nan 250012, China; 2 Key Laboratory of Traditional Chinese Medicine Classical Theory, Ministry of Education, Shandong University of Traditional Chinese Medicine, Ji’nan 250355, China; 3 Experimental Center, Shandong University of Traditional Chinese Medicine, Ji’nan 250355, China; 4 Department of Encephalopathy, Affiliated Hospital of Shandong University of Traditional Chinese Medicine, Ji’nan 250011, China; 5 Department of Gynecology, Affiliated Hospital of Shandong University of Traditional Chinese Medicine, Ji’nan 250011, China

**Keywords:** premenstrual dysphoric disorder, animal model standardization, estrus cycle, forced swimming test, *Gabra4*

## Abstract

Premenstrual dysphoric disorder (PMDD), a form of premenstrual syndrome (PMS), is a severe health disturbance that affects a patient’s emotions; it is caused by periodic psychological symptoms, and its pathogenesis remains unclear. As depression-like symptoms are found in a majority of clinical cases, a reliable animal model of premenstrual depression is indispensable to understand the pathogenesis. Herein, we describe a novel rat model of premenstrual depression, based on the forced swimming test, with a regular estrous cycle. The results showed that in the estrous cycle, the depression-like behavior of rats occurred in the non-receptive phase and disappeared in the receptive phase. Following ovariectomy, the depression-like symptoms disappeared and returned after a hormone priming regimen. Moreover, fluoxetine, an anti-depressant, could reverse the behavioral symptoms in these model rats with normal estrous cycle. Further, the model rats showed significant changes in the serum levels of estrogen and progesterone, hippocampal levels of allopregnanolone, 5-hydroxytryptamine, norepinephrine, and γ-aminobutyric acid (GABA), and in the expression of GABA_A_ receptor 4α subunit, all of which were reversed to physiological levels by fluoxetine. Overall, we established a reliable and standardized rat model of premenstrual depression, which may facilitate the elucidation of PMS/PMDD pathogenesis and development of related therapies.

## INTRODUCTION

Premenstrual syndrome (PMS) is a common mental health disturbance affecting women of childbearing age. Approximately 5‒8% of patients with PMS manifest severe and debilitating symptoms; they are diagnosed with the severe form of PMS referred to as premenstrual dysphoric disorder (PMDD) [1, 2]. The emotional or physical symptoms of PMDD including irritability, depression, anxiety, and anger generally occur in the premenstrual phase of the menstrual cycle and subside with the end of menstruation [2, 3]. These symptoms, especially depression, severely interfere with work, school, usual social activities, and interpersonal relationships (e.g., avoidance of social activities, decreased productivity and efficiency at work, school, or home); therefore, a better understanding of the etiology and pathogenesis of PMDD is needed.

Clinically, the symptoms of PMS/PMDD have been confirmed to be related to cyclic variations in sex hormones occurring during the luteal phase, and patients commonly experience a much sharper decline in progesterone level at the end of the luteal phase when the depression-like symptoms are the most severe [4–6]. Moreover, these symptoms disappear in some patients following ovariectomy or treatment with oral contraceptives [7–9]. In an attempt to improve the quality of life, some patients used hormones to artificially induce the menstrual cycle [10–12] or discontinued oral contraceptives, which led to symptom recurrence [13]. This evidence suggests a need to establish and verify an animal model of PMDD.

An animal model of PMDD is indispensable to further explore its pathogenesis and for the development of therapies. Although several rodent models of PMDD have been developed, most of the related studies have primarily focused on the anxiety-like behavior or the behavior of anxiety mixed with depression [13–16]. Currently, the progesterone withdrawal paradigm is the most widely used animal model; it replicates some key features of PMDD with long-term exogenous steroid hormone administration, followed by abrupt hormone withdrawal [14–16]. Generally, in the progesterone withdrawal paradigm, progesterone is first intraperitoneally injected for 7 days. Then, these animals are stratified into groups and the drug under study is administered till the end of the experiment. In this period, progesterone is continuously injected for about 21 days, and behavioral experiments and samplings are performed within 72 hours after the progesterone injection. In the abovementioned modeling protocol, drug administration, and testing, the estrous cycle is not taken into account [15]. Obviously, this model fails to simulate the core characteristics of PMS (the estrous cycle-dependent symptoms), which occur in the premenstrual period and disappear in the postmenstrual phase. Additionally, the progesterone withdrawal paradigm cannot be used to reveal or mimic the mechanisms underlying real clinical cases of PMDD. Previous studies reported that specific encephalic regions of some patients are more sensitive to variations in allopregnanolone level than those of other patients; moreover, only some of the patients, but not all, developed severe symptoms of PMDD [5, 17–19]. In light of these limitations of the progesterone withdrawal paradigm, researchers have developed newer animal models of PMDD using different behavioral paradigms to mimic the mechanism underlying real cases of PMDD. These include animal models that mainly manifest anxiety-like behaviors, such as resident–intruder [20] and burying harmless objects [21]. In addition, models that mainly show depression-like behaviors have also been developed; to date, only two studies have reported the use of a forced swim-based model of premenstrual depression [22, 23]. However, information on the systematic and precise working of this model, such as the neurochemical and central nervous system mechanisms, is lacking. As depression is a hallmark symptom of PMDD, the establishment of a reliable PMDD model that primarily manifests depression-like symptoms is essential; such a model will likely assist both the exploration of the mechanism of PMS/PMDD under conditions of depressive symptoms and the development of relevant therapies.

**Figure 1:**
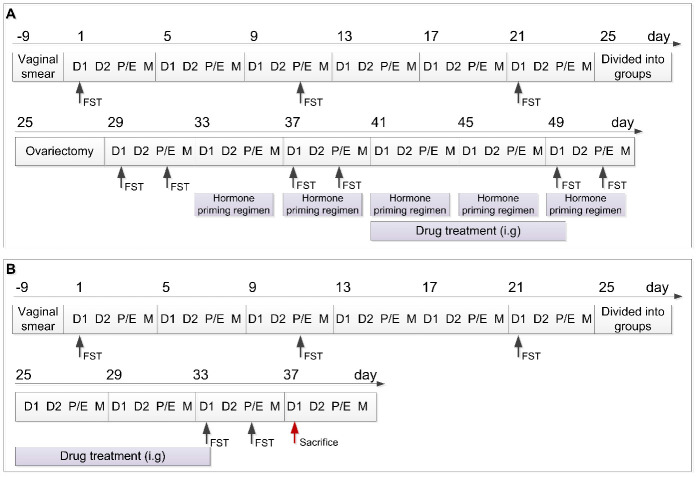
**Schedule of the experimental design including the two experimental parts.** (**A**) Schedule of experimental part 1. (**B**) Schedule of experimental part 2. D1, Diestrus 1 phase; D2, Diestrus 2 phase; P/E, Proestrus/Estrus phase; M, Metestrus phase; FST, Forced swimming test; i.g., intragastrically.

This study aimed to establish a rat model of premenstrual depression, based on the forced swimming test (FST), with a regular estrous cycle. Following ovariectomy and a hormone priming regimen, we assessed depression-like behavior to verify the relationship between the depression-like symptoms and the menstrual cycle. Using the anti-depressant fluoxetine, we conducted a pharmacodynamic effect analysis via a behavioral test in this premenstrual depression model (Figure 1A). In addition, several studies have previously shown that PMDD symptoms in animal models are caused by a decrease in progesterone levels; this consequently causes a change in monoamine neuro-transmitter levels and upregulation of γ-aminobutyric acid A receptor (GABA_A_R) subunit expression in the central nervous system [1, 4, 24–27]. Therefore, this study also assessed the serum levels of estrogen (E_2_) and progesterone, the hippocampal levels of 5-hydroxytryptamine (5-HT), norepinephrine (NE), γ-aminobutyric acid (GABA), and allopregnanolone (ALLO), and the expression of GABA_A_R subunit alpha 4 (GABRA4) in the hippocampus of rats in the control, model, and model + fluoxetine groups (Figure 1B). Through this comprehensive approach, a reliable rat model of premenstrual depression was established and assessed.

## RESULTS

### Evaluation of the rat model of premenstrual depression by FST

### Results of the FST after model establishment and screening

**Figure 2:**
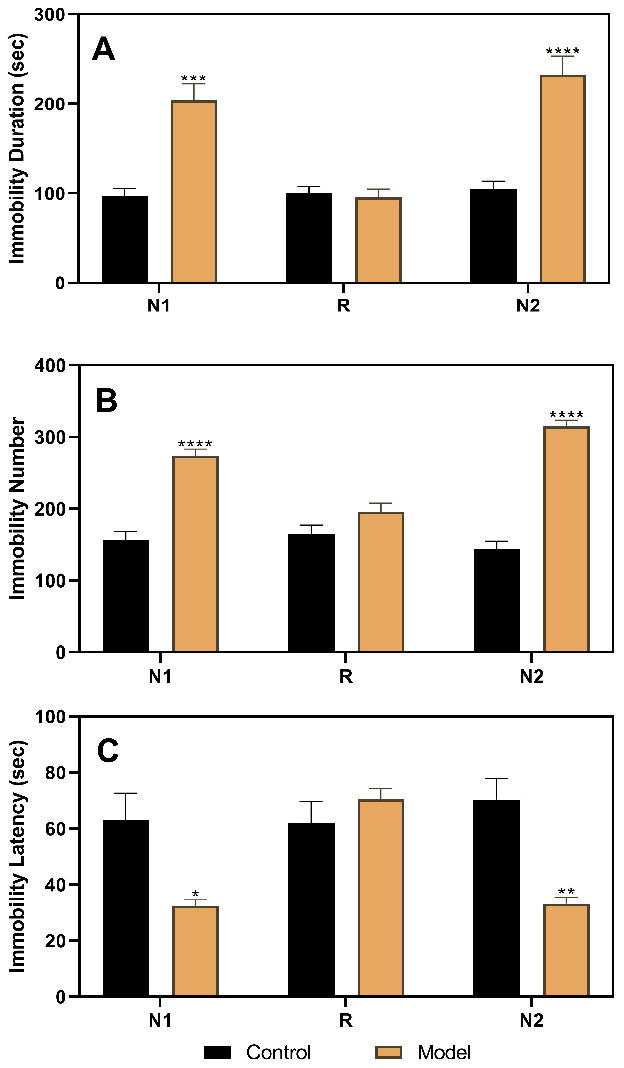
**Results of the forced swimming test after model establishment and screening.** (**A**) Results of immobility duration. (**B**) Results of immobility number. (**C**) Results of immobility latency. N1, the first test in the nonreceptive phase; N2, the second test in the non-receptive phase; R, the test in the receptive phase. *p<0.05, **p<0.01,***p<0.001, ****p<0.0001 (n=8 in the control group and n=16 in the model group; two-way ANOVA followed by post-hoc Sidak’s multiple comparisons test).

After stratifying the experimental rats into the control and model groups, the results of the three FSTs, including two in the diestrus 1 (D1) phase (the first test in the non-receptive phase [N1] and the second test in the non-receptive phase [N2]) and one in the proestrus/estrus (P/E) phase (receptive phase [R]), were analyzed (Figure 2). The results of the analysis of variance (ANOVA) showed that the group factor had a significant effect on immobility duration (F(1,22)=22.52, *p*<0.0001), immobility number (F(1,22)=107.4, *p*<0.0001), and immobility latency (F(1,22)=14.76, *p*=0.0009). In the N1 and N2 tests, rats in the model group (n=16) had a longer immobility duration (N1, p=0.0001; N2, *p*<0.0001), higher immobility number (N1, *p*<0.0001; N2, *p*<0.0001), and shorter immobility latency (N1, *p*=0.0434; N2, *p*=0.0037) than those in the control group (n=8). However, both groups showed no differences in the R phase.

### Results of the FST after ovariectomy and hormone priming regimen

**Figure 3:**
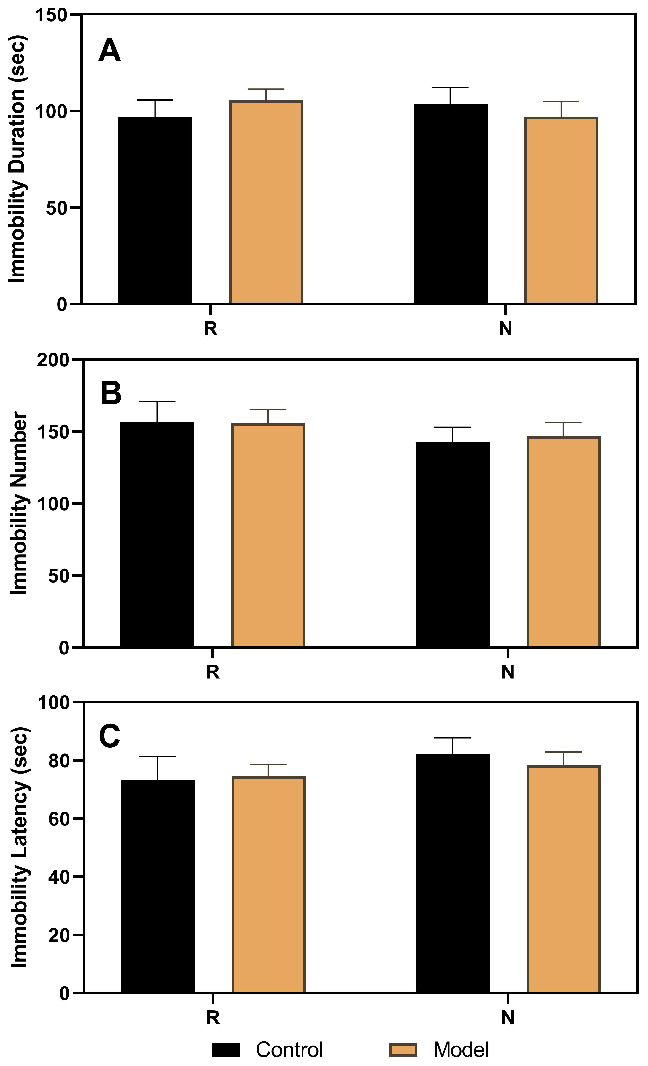
**Results of the forced swimming test after ovariectomy.** (**A**) Results of immobility duration. (**B**) Results of immobility number. (**C**) Results of immobility latency. N, the test in the non-receptive phase; R, the test in the receptive phase (n=8 in the control group and n=16 in the model group; two-way ANOVA followed by post-hoc Sidak’s multiple comparisons test).

**Figure 4:**
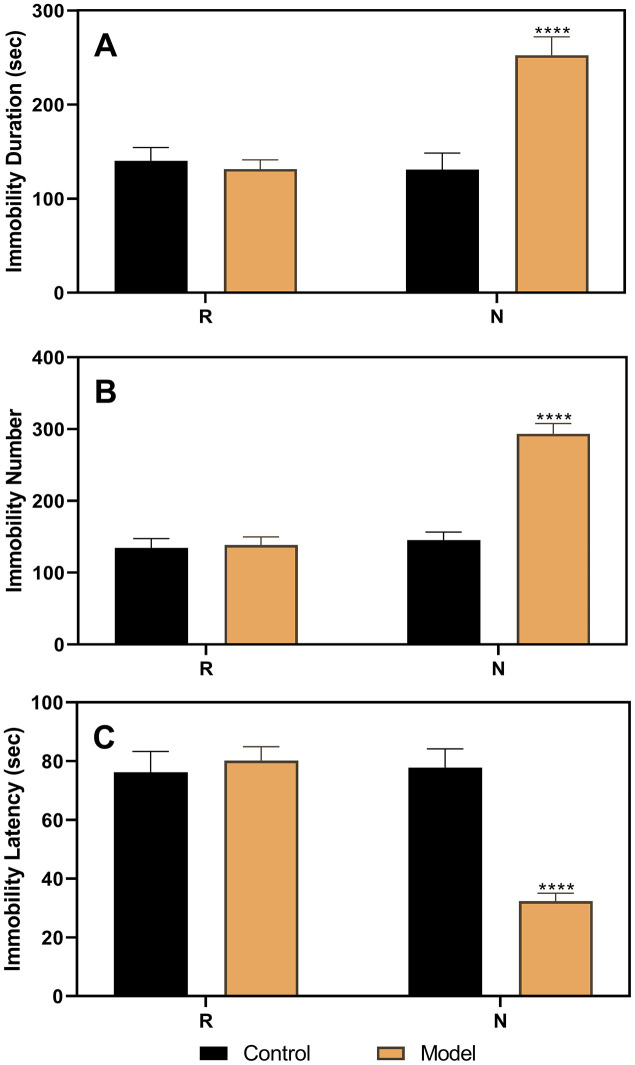
**Results of the forced swimming test after the hormone priming regimen.** (**A**) Results of immobility duration. (**B**) Results of immobility number. (**C**) Results of immobility latency. N, the test in the non-receptive phase; R, the test in the receptive phase. ****p<0.0001 (n=8 in the control group and n=16 in the model group; two-way ANOVA followed by post-hoc Sidak’s multiple comparisons test).

As shown in Figure 3, the results of the FST performed in ovariectomized rats revealed no differences between the control and model groups. However, after the hormone priming regimen (Figure 4), significant differences were detected in the non-receptive phase but not in the receptive phase. The group factor had an obvious effect on the immobility duration in the non-receptive phase (F(1,22)=8.943, *p*=0.0020), and the model group had a longer immobility duration (*p*<0.0001) than the control group. The same effect of group factor was observed for the immobility number (F(1,22)=45.66, *p*<0.0001) with a higher value in the model group (*p*<0.0001) compared to that in the control group. Group factor also had an effect on the immobility latency (F(1,22)=13.91, *p*=0.0012), with the model group showing a longer latency (*p*<0.0001) than the control group.

### Results of the FST after fluoxetine treatment

**Figure 5:**
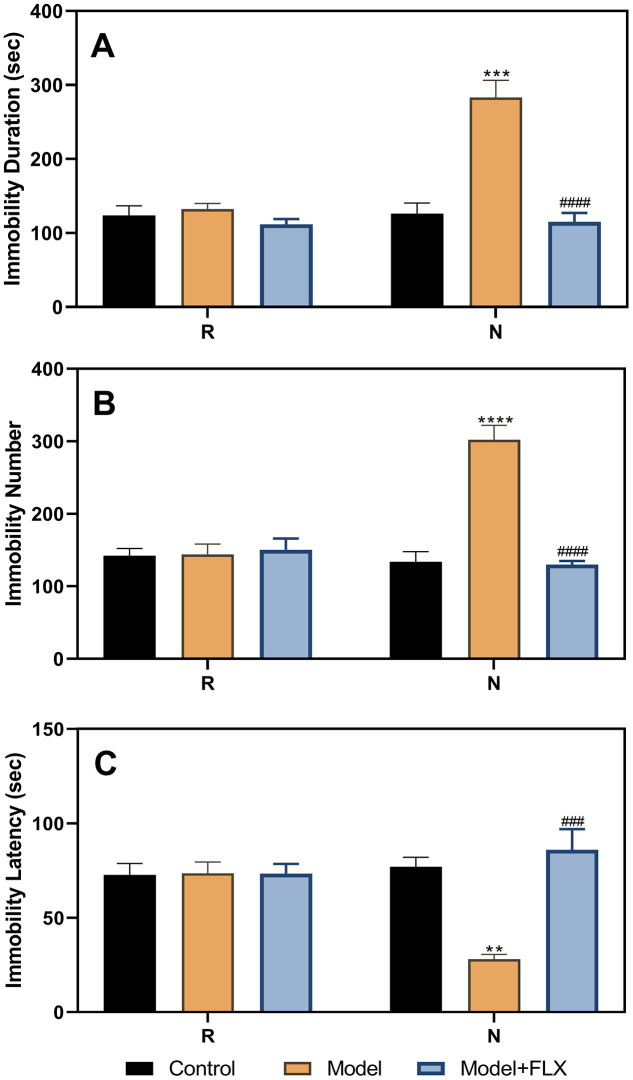
**Results of the forced swimming test after fluoxetine treatment of the hormone-primed rats.** (**A**) Results of immobility duration. (**B**) Results of immobility number. (**C**) Results of immobility latency. N, the test in the non-receptive phase; R, the test in the receptive phase. **p<0.01 compared to the control group, ***p<0.001 compared to the control group, ****p<0.0001 compared to the control group, ###*p*<0.001 compared to the model group, ####*p*<0.0001 compared to the model group (n=8 in each group; two-way ANOVA followed by post-hoc Sidak’s multiple comparisons test).

After administration of fluoxetine to the ovariectomize hormone-primed model animals, the FST was performed again during the non-receptive and receptive phases. As shown in Figure 5, the results of the ANOVA showed significant differences between the different groups (control, model, and model + fluoxetine groups) for immobility duration (F(2,21)=39.14, *p*<0.0001), immobility number (F(2,21)=19.95, *p*<0.0001), and immobility latency (F(2,21)=11.19, *p*=0.0005). Differences between the groups were also observed in the non-receptive phase. The model group (n=8) had a longer immobility duration (*p*=0.0002) than the control group (n=8); however, treatment with fluoxetine reversed this effect (n=8, *p*<0.0001). Similar results were obtained for the immobility number (control vs model, *p*<0.0001; model vs model + fluoxetine, *p*<0.0001). In addition, the model group had a shorter immobility latency than the control group (*p*=0.0039), which improved significantly following fluoxetine treatment (*p*=0.0007).

**Figure 6:**
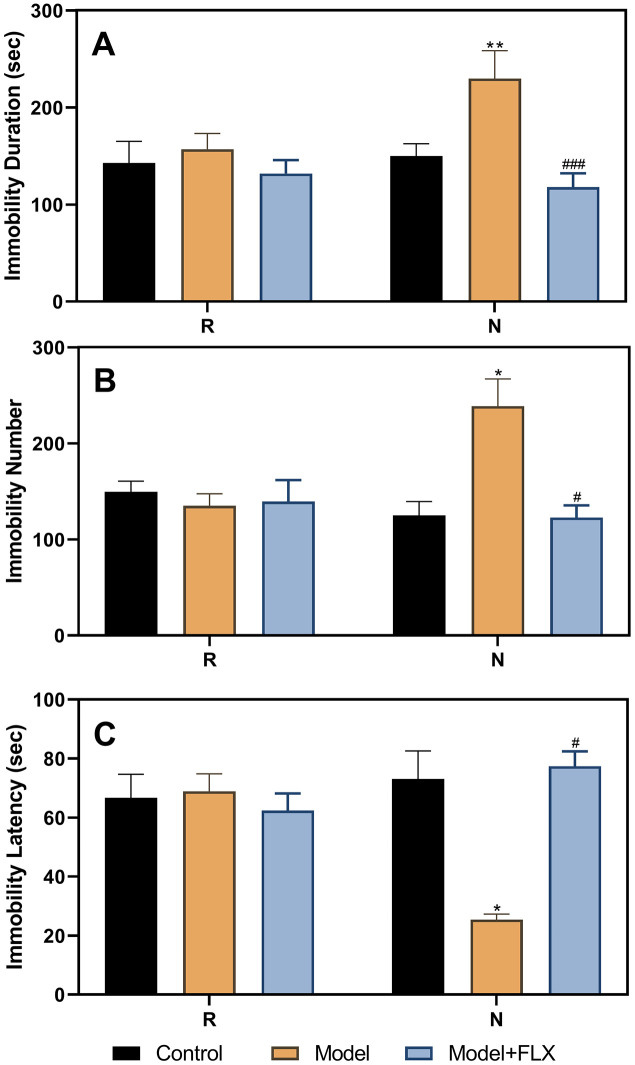
**Results of the forced swimming test after fluoxetine treatment in rats with a normal estrous cycle.** (**A**) Results of immobility duration. (**B**) Results of immobility number. (**C**) Results of immobility latency. N, the test in the non-receptive phase; R, the test in the receptive phase. *p<0.05 compared to control group, **p<0.01 compared to the control group, #*p*<0.05 compared to the model group, ###*p*<0.001 compared to the model group (n=8 in each group; two-way ANOVA followed by post-hoc Sidak’s multiple comparisons test).

We also administered drugs in the model animals with a normal estrous cycle (Figure 6); the results obtained were consistent with those obtained for the ovariectomized, hormone-primed model animals. The group factor had a significant effect on immobility duration (F(2,21)=13.71, *p*=0.0002), immobility number (F(2,21)=5.184, *p*=0.0158), and immobility latency (F(2,21)=6.929, *p*=0.0049). In the non-receptive phase, rats in the model group (n=8) had a longer immobility duration (*p*=0.0056), higher immobility number (*p*=0.0439), and shorter immobility latency (*p*=0.0109) than those in the control group (n=8). Further, rats in the model + fluoxetine (n=8) group had a shorter immobility duration, lower immobility number, and longer immobility latency than those in the model groups (*p*=0.0001, 0.0219, and 0.0109, respectively).

### Changes in E_2_ and neurotransmitters in rats with premenstrual depression

**Figure 7:**
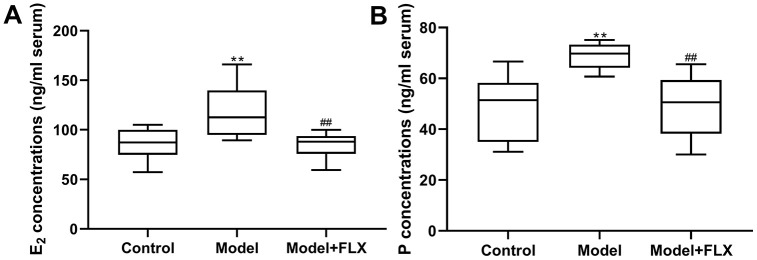
**Estrogen (E2) and progesterone (P) levels in serum**. (**A**) E2 levels in serum. (**B**) P levels in serum. FLX, fluoxetine. **p<0.01 compared to the control group, ##*p*<0.01 compared to the model group (n=8 in each group; one-way ANOVA followed by post-hoc Tukey’s multiple comparisons test).

**Figure 8:**
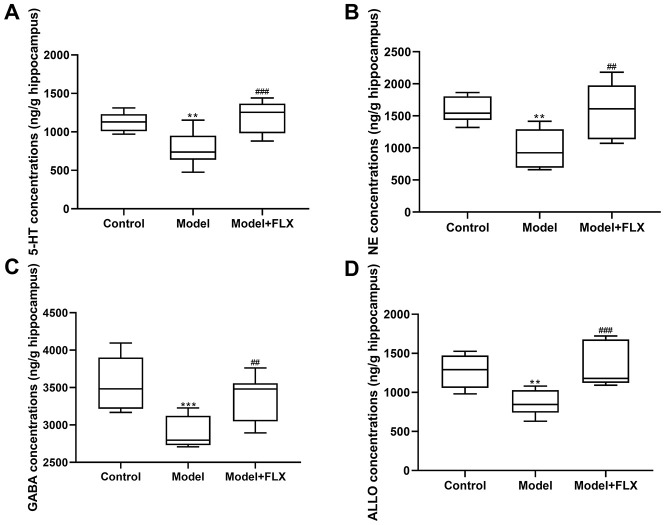
**5-hydroxytryptamine (5-HT), norepinephrine (NE), γ-aminobutyric acid (GABA), and allopregnanolone (ALLO) levels in the hippocampus.** (**A**) 5-HT levels in the hippocampus. (**B**) NE levels in the hippocampus. (**C**) GABA levels in the hippocampus. (**D**) ALLO levels in the hippocampus. FLX, fluoxetine. **p<0.01 compared to the control group, ***p<0.001 compared to the control group, ##*p*<0.01 compared to the model group, ###*p*<0.001 compared to the model group (n=8 in each group; one-way ANOVA followed by post-hoc Tukey’s multiple comparisons test).

Rats in the premenstrual depression model group (n=8) had higher serum levels of E_2_ (F(2,21)=8.044, *p*=0.0025; control vs model *p*=0.0074) and progesterone (F(2, 21)=9.162, *p*=0.0014; control vs model *p*=0.0032) than those in the control group (n=8) in the D1 (non-receptive) phase. These changes in serum E_2_ and progesterone levels could be reversed by fluoxetine treatment (n=8) (*p*=0.0052 and 0.0040, respectively) (Figure 7). Moreover, the hippocampal levels of 5-HT, NE, GABA, and ALLO were detected by an enzyme-linked immunosorbent assay (ELISA). As shown in Figure 8, rats in the model group had lower levels of 5-HT (F(2,21)=10.80, *p*=0.0006; control vs model *p*=0.0039), NE (F(2,21)=10.19, *p*=0.0008; control vs model *p*=0.0021), GABA (F(2,21)=10.31, *p*=0.0008; control vs model p=0.0008), and ALLO (F(2, 21)=10.83, *p*=0.0006; control vs model *p*=0.0041) in the hippocampus than those in the control group. However, all of these changes were reversed in rats in the model + fluoxetine group (*p*=0.0009, 0.0024, 0.0095, and 0.0008, respectively).

### Changes in *Gabra4* expression in rats with premenstrual depression

**Figure 9:**
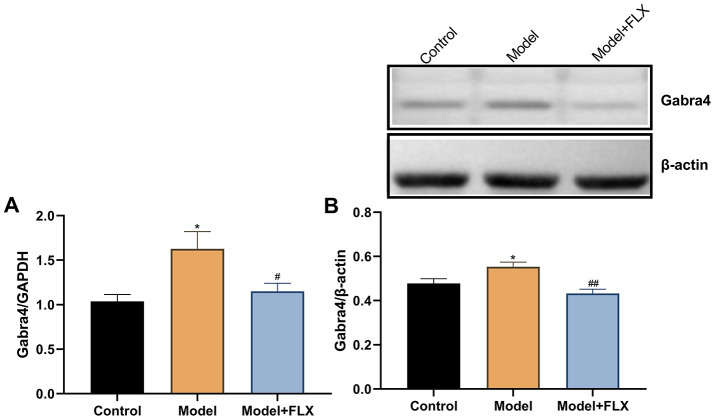
**The mRNA and protein expression levels of GABRA4 in the hippocampus.** (**A**) The mRNA expression levels of GABRA4 in the hippocampus. (**B**) The protein expression levels of GABRA4 in the hippocampus. FLX, fluoxetine. *p<0.05 compared to the control group, #*p*<0.05 compared to the control group, ##*p*<0.01 compared to the model group (n=8 in each group; one-way ANOVA followed by post-hoc Tukey’s multiple comparisons test).

The expression levels of *Gabra4* mRNA and GABRA4 protein were determined by quantitative real-time polymerase chain reaction (RT-qPCR) and western blotting, respectively. As shown in Figure 9, relative to *Gapdh*, the mRNA expression of *Gabra4* (F(2,21)=5.707, *p*=0.0105) was increased in the model group (*p*=0.0120) but reversed to control levels in the model + fluoxetine group (*p*=0.0445). Similar changes were observed for the protein expression level of GABRA4 (relative to β-actin; F(2,21)=8.652, *p*=0.0018; control vs model, p=0.0448; model vs model + fluoxetine, *p*=0.0014).

## DISCUSSION

In this study, a rat model of premenstrual depression, which simulated the symptoms of depression in patients with PMDD, was successfully established based on recent evidence obtained through behavioral symptom assessments by FST and measurements of changes in E2, P, and neurochemical levels. The behavioral symptoms and changes in the levels of disease-related E2, P, neurotransmitters, and GABRA4 could be reversed by treatment with the anti-depressant fluoxetine, thereby verifying the successful establishment of the model in another aspect.

The FST-based rat model of premenstrual depression was established in rats with a regular estrous cycle. As shown in Figure 2, the depression-like behavior occurred in the non-receptive phase and disappeared in the receptive phase of the estrous cycle, consistent with the clinical finding that depression-like symptoms are related to the sharp decline of progesterone at the end of the luteal phase in humans [5, 7]. FST has been used as a method to establish PMS in previous studies [22, 23] and has been found to be reliable for simulating premenstrual depression-like behaviors. However, these studies rarely explored the neurochemical and central nervous system mechanisms underlying the depression-like symptoms, and information on the systematic evaluation of the models was lacking. Behavioral tests that can distinguish estrus cycle-dependent behavioral characteristics are necessary in PMS/PMDD-related research. However, not all behavioral tests meet this requirement; for example, the open field test and sucrose preference test cannot be used to identify behavioral differences in different stages of the estrus cycle [28]. In previous studies, limited behavioral paradigms were used to distinguish these estrus cycle-dependent behavioral changes, such as the resident–intruder test [20], FST [22, 23], and marble burying test [21, 28]. Undoubtedly, the ability of FST to identify these estrus cycle-dependent changes sufficiently enables the detection of PMDD and depression-like phenotypes, as FST is one of the recognized pharmacologic methods to screen for the efficacy of anti-depressive drugs [29]. In this study, the immobility duration, immobility number, and immobility latency were used as indicators of behavioral symptoms, which reflected the depression emotional response, “behavioral despair,” and met the basic requirements of establishing this animal model [30].

To verify the reliability of this animal model of premenstrual depression, we performed a series of related trials. First, we observed that the symptoms of the model rats disappeared following ovariectomy and returned to normalcy after periodic estrus induction with a hormone priming regimen (Figures 3 and 4). These findings demonstrate the relationship between depressive symptoms and ovarian hormone, reflecting the pathogenesis of PMDD [8, 12, 31]. Moreover, in model rats with a hormone-induced estrous cycle (Figure 5) or normal estrous cycle (Figure 6), the first-line anti-depressant fluoxetine could reverse the behavioral symptoms, thereby verifying the accuracy of the symptoms of depression established in this rat model. Although several methods, including progesterone withdrawal, have been widely used to establish animal models of PMS/PMDD [14, 15, 21, 32, 33], they cannot be used to establish a reliable PMDD model that primarily manifests depression-like symptoms. Moreover, these models cannot determine the differences in symptoms between the non-receptive and receptive phases, thereby impeding further research on the improvement of the depression-like symptoms of PMDD. Clinically, in patients with PMS/PMDD, the depression-like symptoms occur in the premenstrual luteal phase and disappear in the follicular phase; thus, the evaluation of the analogous phenomenon in animals is key to the successful establishment of an animal model of PMS/PMDD.

We preliminarily investigated the mechanism underlying PMDD-related depression in relation to progesterone metabolites and neurotransmitters in the central nervous system and evaluated the validity of this model from a deeper perspective. Therefore, the levels of the related hormones (E_2_ and progesterone in serum, and ALLO in the hippocampus), emotion-related neurotransmitters (5-HT, NE, and GABA in the hippocampus), and the expression of GABRA4, which is essential to the pathogenesis of PMS/PMDD [26], were determined (Figures 7‒9). Further, 5-HT and NE play a key role in reconciling the functional neural circuits in the brain and regulating depression-like emotions [34, 35]; moreover, the anti-depressant fluoxetine is a 5-HT reuptake inhibitor. Hence, we considered that exploring the changes in the levels of these neurotransmitters in this premenstrual depression model is indispensable. Our results showed decreased 5-HT and NE levels in the model group, which was reversed by fluoxetine treatment, indicating that levels of monoamine neurotransmitters were abnormally changed in PMDD-related depression. In previous studies, ALLO was regarded as an allosteric agent acting on the GABA A receptors, thereby reducing the GABA-mediated neuronal activities and functions [36]. GABRA4 also reportedly plays important roles in PMS/PMDD [24, 37, 38]. Therefore, we examined ALLO and GABA levels as well as GABRA4 expression levels in the emotion-related brain region, the hippocampus; the results showed decreased ALLO and GABA levels and increased GABRA4 expression levels in the model group, consistent with the viewpoint that GABA-mediated neuronal activity is inhibited in patients with depression [39]. Similarly, the changes in the related hormones, neurotransmitters, and GABRA4 expression were consistent with previous clinical findings. Therefore, these changes might constitute a potential mechanism underlying PMDD-related depression and could serve as reliable evidence for the validity of this animal model.

Several factors may have affected the experimental results obtained in this study. For example, exposure of the experimental rats to the same experimental environment might have led to familiarity, which is also called “habituation” [40, 41]. To avoid “habituation” bias, we used a normal control group for all behavioral experiments. However, the results of FST might have been affected. Further, some behavioral paradigms, such as the marble burying behavior and behaviors on an elevated plus maze, were reported to be estrous cycle-dependent. We repeated the elevated plus maze test several times (data not shown) but failed to prove that the results were estrous cycle-dependent. Therefore, such behavioral paradigms that could identify PMDD depression should be further studied. Lastly, evidence such as metabolic profiling of animal models as well as differences among genes, transcription levels, and protein levels should be provided in the future, which will help support whether these models are effective and reliable.

In conclusion, an animal model of premenstrual depression was evaluated from several aspects and established based on a series of evidence obtained through behavioral tests and biochemical analyses of the central nervous system. Considering the lack of animal models exhibiting depressive symptoms of PMDD, this study may support the further development of a standardized method for establishing a premenstrual depression model, which will facilitate investigations of PMS/PMDD pathogenesis and development of related therapies. As aforementioned, there are several behavioral paradigms that can be used to distinguish estrous cycle-dependent behavioral changes. However, it is unclear whether animal models of PMDD, screened using one behavioral paradigm, also exhibit other estrus cycle-dependent characteristics. To address this caveat, although preliminary attempts have been made by researchers [28], the evidence was not compelling enough. Furthermore, some researchers have shown that the behavior, drug responsiveness, neuronal activity, and related protein expression in the brain of rats or mice with normal estrus cycles seem to be significantly different between the late diestrus phase and the other phases [13, 18, 42, 43]; this observation raised the pertinent question: what is the relationship between the estrous cycle-dependent changes in rats or mice with normal estrus cycles mentioned above and the symptoms of PMDD? This issue requires further research. As the pathogenesis of PMDD is complex, our study only examined some common neurochemical indicators to illustrate the possible mechanism underlying premenstrual depression; thus, deeper mechanistic explorations, such as the evaluation of molecular signaling pathways or neuronal activity recordings in essential brain regions, are lacking herein. Therefore, we are planning a further study in the future to address these queries.

## MATERIALS AND METHODS

### Ethics statement

This investigation was conducted in accordance with the ethical standards and adhered to the tenets of the Declaration of Helsinki, and according to the relevant national and international guidelines; the study was approved by the authors' institutional review board.

### Animals

Female Wistar rats, weighing 135±15 g, procured from the Vital River Laboratories (Beijing, China) were used in this study. The rats were housed at an ambient temperature of 21±1° C and 55% relative humidity with a 12 h/12 h light/dark cycle with day-night reversal (lights on at 20:00; lights off at 8:00). All procedures were conducted under dim light (<25 lux) [44], and food and water were available *ad libitum*. The animals were allowed to acclimate for 1 week before the experiments. All efforts were made to minimize animal suffering.

### Vaginal smear

After acclimation, vaginal smears were obtained from all rats to screen for those with a regular estrus cycle. The estrus cycle of rats includes four phases: proestrus/estrus (P/E, 1 day), metestrus (M, 1 day), diestrus 1 (D1, 1 day), and diestrus 2 (D2, 1 day) [45]. The various estrus cycle phases were confirmed by observing the morphology and proportion of keratinocytes, nuclear epithelial cells, and leucocytes via analysis of the vaginal smears under a microscope (Eclipse C1, Nikon, Japan) [46]. The smear examination was conducted at 09:00 every day for 9 days, and only rats with a regular estrus cycle were used for further experiments.

### FST

This study used FST to establish the rat model of premenstrual depression and to evaluate the depression-like behavior [47, 48]. Rats were placed in plexiglass cylinders that were 20 cm in diameter containing water with a depth of 30 cm and temperature of 23° C for 10 min. The accumulated immobility duration, immobility number, and immobility latency (time from the start of the experiment to the first time of immobility) were calculated. The immobility state of animals was defined as no movement except those necessary to keep the nose above water for breathing [15]. The water was changed and the cylinders were cleaned carefully after each test. This test was performed between 12:00 and 16:00 [49].

### Grouping

As shown in Figure 1, FST was performed on the 1^st^ (D1 phase of the first estrus cycle), 11^th^ (P/E phase of the second estrus cycle), and 21^st^ (D1 phase of the third estrus cycle) day of the experimental schedule. Rats were ordered from high to low according to the difference in duration between the immobility duration in the P/E phase and the immobility duration in the D1 phase. Rats in the top 30% of the orders were randomly stratified into the model group and model + fluoxetine group, and rats in the bottom 30% were assigned to the control group [28].

### Hormone priming regimen

To verify the relationship between depression-like behavior and E_2_ expression in the estrus cycle, rats were subjected to ovariectomy on the 25^th^ day of the experimental schedule under anesthesia with 2% pentobarbital sodium (60 mg/kg) [50]. After recovery for 3 days, FST was performed during the D1 and P/E phases of the next estrus cycle (29^th^ and 31^st^ day, respectively). Next, exogenous E_2_ was administered to induce periodic estrus according to the following regimen: rats were injected with 0.5 μg estradiol benzoate (McLean, China; 10042617) on the day of the D1 phase, 0.5 μg estradiol (McLean, China; 10006920) on the day of the D2 phase, and 0.5 mg progesterone (Sigma, United States; 101327062) on the day of the P/E phase [20]. These estrogens were dissolved in 0.1 mL saline and injected subcutaneously at 12:00 each day. For the second cycle of the hormone priming regimen, FST was performed on the 37^th^ day (D1) and 39^th^ day (P/E), respectively. The hormone priming regimen was administered until the end of the experiment in Part 1.

### Drug treatment

In Part 1 of the experimental schedule, fluoxetine was administered to the rats in the model + fluoxetine group from the 41^st^ to the 49^th^ day. Dispersible tablets of fluoxetine (2.7 mg/kg/d; Lilly Company, China; H20050463) were dissolved in saline (2.7 mg/mL) and administered intragastrically at 08:00 each day. Rats in the control and model groups received the same administration with saline. After the drug treatment, FST was performed on the 49^th^ (D1) and 51^st^ (P/E) day, respectively. In Part 2 of the experimental schedule, the same drug regimen was administered after the grouping on the 25^th^ day of the schedule for 9 days (to the 33^rd^ day), and then FST was performed on the 33^rd^ (D1) and 35^th^ (P/E) day, respectively.

### ELISA

After FST, the rats in Part 2 of the experiment were anesthetized with an overdose of 2% pentobarbital sodium and killed by cervical dislocation on the 37^th^ day (D1 phase) of the schedule, and then 5 mL of peripheral blood was collected and centrifuged at 3000 r/min (4 ° C) for 15 min. The plasma was separated and frozen at -80° C for further ELISA analysis of E_2_ and progesterone levels. Simultaneously, the brains of the culled rats were removed, and the hippocampi were isolated on ice. Samples from the left hippocampi were used for ELISA analysis of GABA, 5-HT, NE, and ALLO, and those from the right hippocampi were used for western blot and PCR analyses of the GABRA4 subunit. The brain tissue for ELISA was homogenized in phosphate buffer, and then the supernatant was collected by centrifugation at 13,000 r/min (4° C) for 10 min for further analysis. Next, the levels of E_2_ and progesterone in the serum and those of GABA, 5-HT, NE, and ALLO in the hippocampus were measured using ELISA kits (Cusabio, China) as previously described [49]. Thereafter, the optical density was determined using a microplate spectrophotometer at 450 nm.

### Western blot analysis

The right hippocampus tissues were grounded in liquid nitrogen and homogenized in extraction buffer (Solarbio, China; R0010). After ultra-sonification for 10 min, the sample was centrifuged at 12,000 r/min (4° C) for 30 min, and then the supernatant was collected. Thereafter, protein levels were determined at 570 nm using a bicinchoninic acid protein assay kit (Solarbio, China; PC0020) according to the manufacturer’s instructions. Samples (20 μL) were then loaded onto a 10% sodium dodecyl sulfate–polyacrylamide gel after boiling for 5 min and then separated by electrophoresis. The separated proteins were then electrophoretically transferred to a polyvinylidene difluoride membrane and blocked in 5% non-fat milk at 25° C for 1 h. The membrane was incubated with primary antibodies (1:200 rabbit polyclonal anti-GABRA4 antibody; OriGene, China; TA328815, and 1:800 mouse polyclonal anti-β-actin antibody; Sungene Biotech, China; KM9001) and then incubated with the secondary antibody (1:5000 horseradish peroxidase-conjugated Affinipure Goat Anti-Rabbit/mouse IgG; Proteintech, China; SA00001-1/2). The positive protein bands were visualized using an enhanced chemiluminescence reagent purchased from Solarbio (China; PE0010), and then the optical density value was calculated on the Image J Software. β-actin was used as the internal reference, and the GABRA4/β-actin ratio was used to evaluate the protein levels.

### RT-qPCR analysis

Total RNA was extracted from hippocampal tissue using 1 mL Trizol reagent (Sigma, United States) and reversed-transcribed to cDNA using ReverTra Ace qPCR RT Master Mix (TOYOBO, Japan; FSQ-201). RT-qPCR was performed using THUNDER BIRD SYBR qPCR Mix (TOYOBO, Japan; FSQ-101) with the Roche LightCycler 480 Real-Time PCR System. Data analysis was performed using the Light Cycle96 SW1.1 Software by the 2^-ΔΔCt^ method; the mRNA expression of glyceraldehyde-3-phosphate dehydrogenase (*Gapdh*) was used for normalization. The relative gene expression (*Gabra4*/*Gapdh*) was calculated and used for statistical analysis. The primers used for RT-qPCR were synthesized by Sangon Biotech (China) and the sequences were as follows: *Gabra4*-Forward primer: 5′-GAAACCACTCCTAAGGCCCACT-3′ Gabra4-Reversed primer: 5′- GCGATGCGGCAGACGAAA -3′ *GAPDH*-Forward primer: 5′-GGCAAGTTCAACGGCACAGT-3′*GAPDH*-Reversed primer: 5′-TGGTGAAGACGCCAGTAGACTC-3′.

### Statistical analysis

Data analysis was performed using GraphPad Prism 8.0.2, and the data are expressed as mean ± standard error of the mean. For analysis of normality of distribution, the non-parametric Kolmogorov–Smirnov test was applied. Using the modified Layida method, exceptional data exceeding the mean ± 2× the standard deviation were removed [51]. Differences in the FST results were compared using two-way ANOVA followed by post-hoc Sidak’s multiple comparisons test. For the neurochemical and biochemical data, one-way ANOVA with post-hoc Tukey’s multiple comparisons test was used. The level of significance was set at p < 0.05.
